# Hematological and Biochemical Parameters of European Quails (*Coturnix coturnix coturnix*) Fed Diets with Different Fiber Profiles, with and Without Stimbiotic Inclusion, from 1 to 35 Days

**DOI:** 10.3390/ani15233457

**Published:** 2025-11-30

**Authors:** Luayne Morais Correa, Adiel Vieira de Lima, Edijanio Galdino da Silva, Anderson Antonio Ferreira da Silva, Maria das Graças da Silva Bernardino, Raiane dos Santos Silva, Neila Lidiany Ribeiro, Jammily Ketly Guedes Caetano, Xavière Rousseau, Matheus Ramalho de Lima, Fernando Guilherme Perazzo Costa, Ricardo Romão Guerra

**Affiliations:** 1 Department of Veterinary Sciences, Center of Agricultural Sciences, Federal University of Paraiba, Areia 58397-000, Paraiba, Brazil; luaynemoraes@icloud.com (L.M.C.); edijanio@veterinario.med.br (E.G.d.S.); bernardino.mgs@outlook.com (M.d.G.d.S.B.); jammily.ketly1@gmail.com (J.K.G.C.); 2 Department of Animal Science, Center of Agricultural Sciences, Federal University of Paraiba, Areia 58397-000, Paraiba, Brazil; adiel1205@hotmail.com (A.V.d.L.); andersonzootec@hotmail.com (A.A.F.d.S.); raianee.saantos@gmail.com (R.d.S.S.); neilalr@hotmail.com (N.L.R.); 3 Ab Vista, 3 Woodstock Court, Marlborough, Wiltshire SN8 4AN, UK; xaviere.rousseau@abvista.com; 4 Animal Science Departament, Federal University of the Semi-Arid Region, Campus Mossoró, Mossoro 59625-900, Rio Grande do Norte, Brazil; mrlmatheus@ufersa.edu.br

**Keywords:** serum biochemistry, hematology, immunity, metabolism, nutrition

## Abstract

Quail farming necessitates the provision of balanced diets to promote avian health and performance, with dietary fiber and feed additives exerting significant influence on immune and physiological responses. This study aimed to evaluate the effects of stimbiotic inclusion in diets characterized by varying fiber profiles on hematological and biochemical parameters in European quails (*Coturnix coturnix coturnix*). A total of six hundred quails, aged from 1 to 35 days, were assigned to 12 distinct treatments, each comprising 5 replicates of 10 birds. The dietary treatments included a corn–soy control, two basal diets featuring corn (low soluble fiber) and wheat (high soluble fiber), and three mixed diets that combined corn and wheat in varying proportions, with each diet supplemented with or without 0.01% stimbiotic. Blood samples were collected at 14 and 35 days of age. In the initial phase (1–14 days), the addition of stimbiotic resulted in increased hemoglobin levels and a reduction in total white blood cells, heterophils, and lymphocytes, indicative of an enhanced immune status. In contrast, dietary fiber was found to influence liver enzyme activity and triglyceride levels. During the growth phase (15–35 days), stimbiotic continued to exert positive effects on hemoglobin and packed cell volume, while dietary fiber increased eosinophil counts, modulating the immune response without affecting other parameters. In conclusion, dietary fiber plays a functional role, particularly during the growth phase, while stimbiotic inclusion enhances immune function in both developmental stages, demonstrating that both strategies contribute positively to the health and performance of European quails.

## Simple Summary

Quail farming represents an efficient agricultural system that produces high-quality meat and eggs; however, it necessitates the provision of balanced diets to maintain avian health and well-being. Fiber, once deemed detrimental due to the belief that it hinders nutrient absorption, is now acknowledged for its beneficial effects on birds. It facilitates proper gastrointestinal function, supports beneficial microbiota, and enhances overall intestinal health. Feed additives known as stimbiotics, derived from natural enzymes and oligosaccharides, can amplify the advantages of fiber and bolster the immune system of the birds. This study investigated various types of fiber in the diets of European quails, both with and without the inclusion of stimbiotics, to evaluate their impact on hematological health and immune response. The results indicated that stimbiotics enhanced immune responses during both early and later growth stages, while different fiber types exerted a more pronounced effect during the later growth stage, thereby supporting the birds’ innate defenses without altering other indicators of blood health. These findings suggest that both fiber and stimbiotics contribute to the health and productivity of quails, providing valuable strategies for more sustainable quail farming practices.

## 1. Introduction

Quail represent a poultry species of significant economic importance due to the superior quality of their meat, the high nutritional value of their eggs, and distinctive biological characteristics—such as early sexual maturity, elevated laying rates, and rapid growth—which render their farming a fast-return investment requiring minimal space and feed [1]. The performance and health of these birds are contingent upon balanced diets, particularly during the initial (1–14 days) and growing (15–35 days) phases, when the gastrointestinal tract is still undergoing development. During these stages, nutritional requirements diverge from those of other poultry, characterized by a heightened demand for protein, a reduced requirement for calcium, and an increased need for energy [2]. These specific nutritional needs, in conjunction with genetic factors (breed and strain), sex, production purpose (meat or eggs), dietary energy density, nutrient bioavailability, and environmental and sanitary conditions, underscore the necessity for tailored nutritional recommendations for quail [3,4].

Corn and wheat serve as the primary cereal grains utilized in poultry diets as energy sources, owing to their high starch content. However, these grains exhibit substantial differences in the content and composition of non-starch polysaccharides (NSPs). For example, wheat contains a higher total NSP content, encompassing both soluble and insoluble fractions, compared to corn [3]. The NSP profile of wheat is predominantly composed of arabinoxylans, whereas corn contains a greater proportion of cellulose and lower levels of soluble arabinoxylans [5].

Historically, dietary fiber was regarded as a diluting or antinutritional factor due to the presence of NSPs [6,7]. Nevertheless, recent years have seen a paradigm shift, with increasing recognition of its beneficial effects on gut health [8,9]. Since NSPs are indigestible by poultry enzymes, they undergo fermentation by the gut microbiota, resulting in the production of short-chain fatty acids that promote the proliferation of beneficial bacteria [9,10,11,12,13,14,15].

In this context, stimbiotic emerges as a promising technological alternative. Comprising xylanase and xylo-oligosaccharides, it acts synergistically to enhance the degradation of non-starch polysaccharides and to stimulate beneficial microbial fermentation in the gut. This process increases the production of short-chain fatty acids, contributing to improved intestinal health, nutrient utilization, and immune function [16,17,18]. Previous studies in broilers and pigs have shown positive effects of stimbiotic inclusion on growth performance, intestinal morphology, and microbiota balance. However, little is known about its physiological and metabolic effects in quails, particularly when combined with diets differing in fiber profile, which represents a critical knowledge gap that this study aims to address.

The analysis of hematological and biochemical parameters is essential for evaluating animal health. These parameters reflect the physiological state of the animal, providing critical insights into organ function, the animal’s capacity to adapt to nutritional and environmental challenges, and are indispensable for identifying metabolic imbalances and various pathologies [19,20].

Recent studies have demonstrated that stimbiotic inclusion positively influences gut health, productive performance, and the microbiota of broilers [9,14,15,21,22]. However, no research has assessed these effects in meat quail, nor have investigations examined their impacts on hematological and biochemical parameters, particularly during the initial and growing phases. This omission is significant, as inclusion and the definition of diverse fiber profiles directly affect the health, welfare, and physiological responses of the birds.

Consequently, this study aimed to evaluate the effects of different dietary fiber profiles and stimbiotic inclusion on hematological and biochemical parameters of European quails from 1 to 35 days of age, to better understand how these nutritional factors influence metabolism and physiological responses.

## 2. Materials and Methods

### 2.1. Experimental Site and Ethics Committee

The experiment was conducted at the Poultry Experimental Module of the Center for Agricultural Sciences, at the Federal University of Paraíba—Campus II. A total of 720 one-day-old European quail (*Coturnix coturnix coturnix*) with an average weight of 10.17 ± 0.02 g were used. The experiment was submitted to the Animal Use Committee of the Federal University of Paraíba (CEUA-UFPB) under protocol No. 4639170425.

### 2.2. Housing

The treatments were organized in a 6 × 2 factorial design, comprising six fiber profiles and two levels of stimbiotic. The birds were housed in galvanized wire cages with dimensions of 100 × 33.3 × 20 cm (length × width × height), which were equipped with trough-type feeders and nipple drinkers suitable for the developmental stage of the birds. The feeders were replenished with the experimental diets bi-daily at 7:00 a.m. and 4:00 p.m. Throughout the duration of the experimental period, the birds had ad libitum access to both feed and water.

### 2.3. Animals, Experimental Design and Experimental Diets

**Figure 1:**
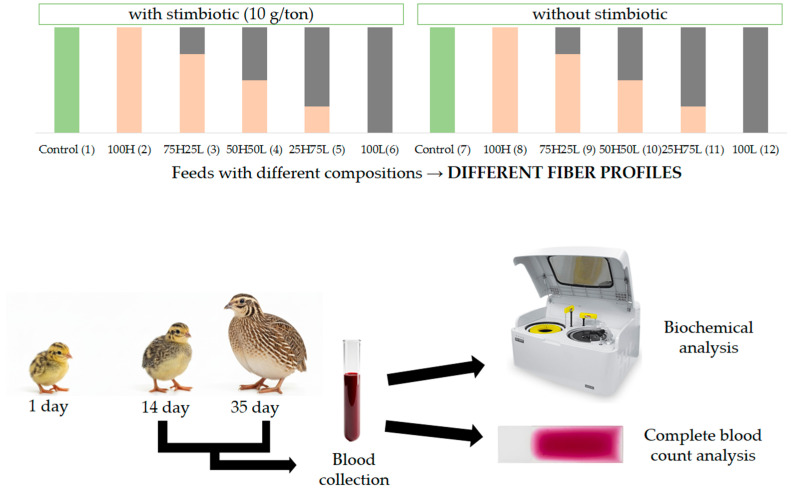
Formation of the experimental treatments from three basal diets (control and high and low fiber), with and without stimbiotic, resulting in 12 experimental treatments, and a schematic representation of blood collection and analyses.

A total of 720 one-day-old European quail (*Coturnix coturnix coturnix*), with an average initial body weight of 10.17 ± 0.02 g, were utilized in this study and allocated in a completely randomized design (CRD) comprising 12 treatments, each replicated six times with 10 birds per replicate. The treatments were designed to facilitate the comparison of various soluble fiber profiles within the diet. Initially, a control diet, consisting of corn and soybean meal, was employed, representing the conventional standard in quail nutrition. Following this, two distinct basal diets were introduced: one exclusively formulated with corn, characterized by a low soluble fiber content (L100), and another composed solely of wheat, which is noted for its elevated soluble fiber content (H100). To assess intermediate effects, three mixed diets were developed by progressively blending wheat and corn in the ratios of L75:H25, L50:H50, and L25:H75, respectively. All diets were formulated in accordance with the recommendations set forth by Silva and Costa [23] (Table 1 and Table 2). The chemical composition of the ingredients utilized in the formulation of the experimental diets was analyzed using Near-Infrared Spectroscopy (NIRS). The six diets exhibiting different fiber profiles were administered with or without the addition of a stimbiotic (Signis, β-1,4-endo-xylanase, and xylo-oligosaccharides, AB Vista, Marlborough, Wiltshire, UK), supplemented at a rate of 100 g/ton of feed, resulting in a total of 12 dietary treatments (Figure 1).

### 2.4. Data Collection

The experiment was conducted in two distinct phases: the initial phase (days 1 to 14) and the growth phase (days 15 to 35). Hematological and biochemical analyses were performed during both phases. On days 14 and 35 of life, eight birds from each treatment group were selected for blood collection, with selection based on the average body weight of the respective treatments. Prior to blood collection, the birds were subjected to a fasting period of six hours. Blood samples were acquired through jugular puncture using a 13 × 0.4 mm needle in syringes containing EDTA. The samples were individually stored in test tubes for subsequent hematological analyses. For leukocyte counting and differential analysis, blood smears were prepared immediately following collection from each bird (see Figure 1).

### 2.5. Collection of Biological Material and Laboratory Analyses

For the assessment of biochemical parameters, blood samples were collected in plain tubes, which were allowed to rest for 30 min before being centrifuged at 3500 rpm for 1 min to isolate the serum. The serum was subsequently stored in Eppendorf tubes and frozen for future analysis.

Biochemical analyses, along with the staining and examination of blood smears, were conducted at the Poultry Laboratory of the Federal University of Paraíba. For biochemical determinations, commercial kits (Labtest Diagnóstica S.A.^®^, Lagoa Santa, Brazil) were utilized to measure aspartate aminotransferase (AST), alanine aminotransferase (ALT), gamma-glutamyl transferase (GGT), proteins, triglycerides, and cholesterol, employing the SINNOWA automatic biochemical analyzer (SX-260, Ribeirão Preto, São Paulo, Brazil). Blood smears were stained using two routine stains, Dift-Quick and May–Grünwald–Giemsa, facilitating differential leukocyte counting and the analysis of potential morphological alterations.

Immediately following collection, blood smears were prepared, air-dried, and subsequently stained with May–Grünwald–Giemsa. Packed cell volume (PCV) was determined using the microhematocrit method [24], while hemoglobin concentration was measured in accordance with the methods established by Weiss and Wardrop [25]. Hematimetric indices, following Wintrobe’s methodology, including mean corpuscular volume (MCV) and mean corpuscular hemoglobin concentration (MCHC), were calculated using established mathematical equations [26]. Total erythrocyte, leukocyte, and thrombocyte counts were performed manually in a Neubauer chamber, utilizing blood diluted in 0.01% toluidine blue at a ratio of 1:200 [27]. Differential leukocyte counts, morphological evaluation of blood cells, and screening for hemoparasites were conducted through examination of the blood smear under a light microscope at 1000× magnification. The heterophil-to-lymphocyte ratio (H:L) was determined according to the methodology described by Onbasilar and Aksoy [28]. All analyses were performed at the Veterinary Hospital of the Center for Agricultural Sciences, Federal University of Paraíba.

### 2.6. Performance Parameters

The performance of the quails was evaluated based on feed intake, weight gain, feed conversion ratio, and carcass yield. The birds and feed residues were weighed at the beginning and at the end of the experimental period (1 to 35 days of age) to determine total feed intake (g/bird) and total weight gain (g/bird), obtained as the difference between the final and initial body weights of the birds and between the feed supplied and the leftovers from each experimental unit. Daily feed intake (g/bird/day) and daily weight gain (g/bird/day) were calculated by dividing the total values by the number of days of the experimental period. The feed conversion ratio (g/g) was calculated as the ratio between total feed intake and total weight gain per experimental unit. At the end of the period, three birds per replicate, with body weight within the group average, were selected for slaughter, fasted for 6 h, and euthanized by electrical stunning followed by bleeding, in accordance with CONCEA guidelines. After evisceration and removal of the edible viscera, the carcasses were chilled at 4 °C for 24 h and then weighed to determine carcass yield (%), calculated as the ratio between carcass weight and live body weight at slaughter, multiplied by 100.

### 2.7. Statistical Analysis

The data were subjected to Analysis of Variance (ANOVA) using R software version 4.2.0 [29] to determine the effects of different fiber profiles (6 levels) and stimbiotic levels (2 levels), as well as their interactions, on the measured variables. For variables showing significant differences (*p* < 0.05), means were compared using Tukey’s test.

The variables were analyzed according to the following mathematical model:

Y_ijk_ = μ + α_i_ + β_j_ + (αβ)_ij_ + ϵ_ijk_

where

- Y_ijk_ = response variable.
- μ = overall mean.
- α_i_ = effect of the i-th fiber profile (i = 1 to 6).
- β_j_ = effect of the j-th stimbiotic level (j = 1 to 2).
- (αβ)_ij_ = interaction effect between the i-th fiber profile and the j-th stimbiotic level
- ϵ_ijk_ = random error term associated with each observation, assumed to be normally distributed with mean zero and constant variance.

## 3. Results

### 3.1. Hematological Parameters

**Figure 2:**
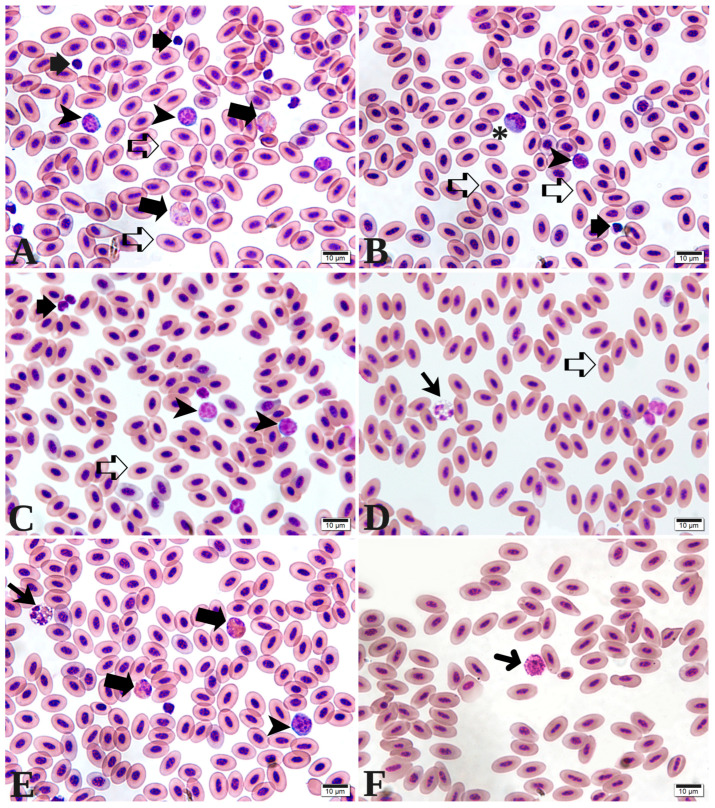
Photomicrographs of blood cells from European quails (*Coturnix coturnix coturnix*). (**A**)—Erythrocytes (open arrow), heterophils (thick arrow), thrombocytes (short arrows), and lymphocytes (arrowhead). (**B**)—Erythrocytes (open arrow), lymphocytes (arrowhead), thrombocytes (short arrows), and monocytes (asterisk). (**C**)—Erythrocytes (open arrow), thrombocytes (short arrows), and lymphocytes (arrowhead). (**D**)—Erythrocytes (open arrows) and eosinophils (arrow). (**E**)—Eosinophils (arrow), heterophils (thick arrow), and lymphocyte (arrowhead). (**F**)—Basophils (thin arrow). Staining: May–Grünwald–Giemsa. Scale bars: (**A**–**F**), 10 µm.

In relation to the hematological parameters, Table 3 illustrates that during the initial phase (from the 1st to the 14th day of life), no significant interaction between fiber and stimbiotic was detected for any of the analyzed variables. The parameters including packed cell volume, erythrocyte count, mean corpuscular volume (MCV), mean corpuscular hemoglobin concentration (MCHC), and components of the white blood cell series (as depicted in Figure 2)—namely eosinophils, basophils, monocytes, and the heterophil/lymphocyte ratio—demonstrated no statistically significant differences attributable to fiber or stimbiotic inclusion. Conversely, hemoglobin, total leukocyte count, heterophils, and lymphocytes were influenced by the levels of stimbiotic incorporated into the diet. Specifically, hemoglobin levels were elevated in animals receiving stimbiotic, while total leukocyte count, heterophils, and lymphocytes were reduced.

Platelet parameters exhibited significant variations in response to fiber inclusion. It was observed that higher fiber content corresponded with reduced platelet counts, whereas diets with lower fiber levels yielded higher platelet counts.

Table 4 presents the hematological values for the growth phase (15 to 35 days of age), revealing no significant interaction between fiber and stimbiotic for any of the hematological variables. However, stimbiotic inclusion significantly enhanced packed cell volume and hemoglobin levels while concurrently decreasing total leukocyte count and lymphocyte counts. With respect to fiber, only eosinophils exhibited significant variation: birds on the control diet presented the lowest eosinophil counts, while those consuming the diet with the highest fiber content (H100) displayed the highest eosinophil values.

### 3.2. Biochemical Parameters

**Figure 3:**
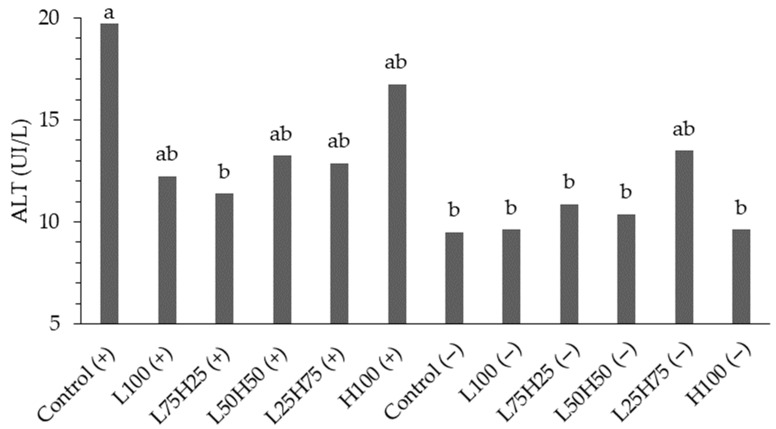
Alanine aminotransferase (ALT, IU/L) levels in European quail fed different fiber profiles, with (indicated by +) or without the inclusion of a symbiotic (indicated by –). The fiber profiles are: Control (corn and soybean meal-based diet); L100 (100% high-fiber diet, based on whole wheat and wheat bran); L75H25 (75% high-fiber diet + 25% low-fiber diet); L50H50 (50% high-fiber diet + 50% low-fiber diet); L25H75 (25% high-fiber diet + 75% low-fiber diet); and H100 (100% low-fiber diet, based on corn germ). Different letters indicate significant differences according to Tukey’s test (*p* < 0.05).

In the biochemical analysis of the initial phase (Table 5), a significant interaction between stimbiotic and fiber was observed concerning alanine aminotransferase (ALT), as illustrated in Figure 3. The evaluation of ALT levels revealed significant differences among the treatments. Birds in the control group that received the stimbiotic exhibited the highest ALT concentration at 19.75 IU/L, which was statistically greater than that of most other treatments. In fiber profiles combined with stimbiotic, ALT values varied from 11.38 IU/L (L75H25) to 16.75 IU/L (H100), with no significant differences detected among these treatments. Conversely, treatments lacking stimbiotic displayed lower and more homogeneous ALT levels, ranging from 9.50 IU/L (control) to 13.50 IU/L (L25H75), with no statistically significant differences between fiber profiles, except for the L25H75 treatment, which demonstrated an intermediate value.

The variables albumin, aspartate aminotransferase (AST), cholesterol, gamma-glutamyl transferase (GGT), and total proteins did not exhibit significant differences. In contrast, triglyceride levels were significantly influenced by both stimbiotic inclusion and fiber content. Specifically, triglyceride concentrations were elevated with the inclusion of stimbiotics, whereas an inverse relationship was observed with fiber content; higher fiber levels corresponded to lower triglyceride values. During the growth phase, none of the biochemical variables assessed demonstrated statistical significance (see Table 6).

### 3.3. Performance Parameters

The performance results of European quails fed diets with varying fiber profiles, with or without stimbiotic inclusion, are presented in Table 7. No statistically significant differences (*p* > 0.05) were observed in initial body weight, thereby confirming the uniformity of the experimental groups at the outset of the trial. Final body weight and total weight gain were not significantly affected by either stimbiotic inclusion or fiber profile; however, quails fed low-fiber diets (100L) exhibited numerically higher body weights in comparison to those receiving high-fiber diets (100H). Both daily and total feed intake were significantly influenced by the experimental factors (*p* < 0.05), with stimbiotic-supplemented quails demonstrating lower feed intake than their non-supplemented counterparts, and birds on high-fiber diets showing reduced intake relative to those on low-fiber diets. The feed conversion ratio (FCR) was significantly enhanced by stimbiotic inclusion (*p* = 0.0491), reflecting an approximate 9% improvement in feed efficiency. Carcass yield was not significantly affected by the treatments (*p* > 0.05). Collectively, the inclusion of stimbiotic appears to enhance feed efficiency without compromising growth performance, indicating improved nutrient utilization and better adaptation of quails to diets with varying fiber profiles.

## 4. Discussion

Packed cell volume (PCV), also referred to as hematocrit, denotes the proportion of blood volume occupied by erythrocytes and serves as a critical hematological indicator for evaluating tissue oxygenation and hydration status in avian species. During the growth phase (15 to 35 days) of the present study, a statistically significant increase in this variable was observed in groups supplemented with the stimbiotic. This response may be attributed to enhanced efficiency in the absorption of nutrients vital for erythropoiesis, a process potentially facilitated by the intestinal modulation induced by the additive. According to Veluri et al. [30], the incorporation of stimbiotics in broiler diets leads to significant improvements in intestinal morphology, characterized by increased villus height, elevated villus-to-crypt ratio, and stimulation of genes associated with epithelial barrier integrity. Such alterations are instrumental in augmenting the absorption of essential minerals, including iron, phosphorus, and magnesium, which are critical for optimal hematopoietic function. Moreover, as emphasized by Martinez et al. [31], stimbiotics play a role in fostering a more stable and functional intestinal environment, thereby optimizing nutrient digestibility. Consequently, the observed enhancement in PCV may signify a favorable physiological response to inclusion, illustrating the indirect hematological potential of these additives through improved nutritional status.

Concerning hemoglobin levels, a significant increase was observed in both experimental phases among the groups supplemented with stimbiotic, indicating a potential correlation with enhanced iron availability, likely facilitated by improved nutrient absorption attributed to this functional additive. Hemoglobin is a hematological parameter that is directly influenced by iron bioavailability, which is critical for erythrocyte production. According to Thrall et al. [32], the physiological hemoglobin values in quail range from 4.0 to 5.2 g/L. The data obtained in the current study surpassed this range, indicating levels higher than the normal reference values.

Among the variables that remained constant across both phases were hematimetric indices, including mean corpuscular volume (MCV), mean corpuscular hemoglobin concentration (MCHC), eosinophil counts, basophil counts, monocyte counts, platelet counts, and the heterophil-to-lymphocyte ratio (H/L). During the initial phase, the quail were engaged in hematopoietic and immunological development, which may have curtailed the observable physiological effects of stimbiotic inclusion. Furthermore, Garber and Parra [33] indicate that stimbiotics exert their influence indirectly by modulating systemic immunity, primarily through the promotion of a favorable intestinal environment that reduces the activation of the immune axis in birds raised under optimal sanitary conditions. Consequently, the lack of significant alterations in certain hematological variables, as noted in the current study, may not indicate the inefficacy of the stimbiotic, but rather reflect the preservation of a physiologically balanced immunological state, negating the necessity for substantial hematopoietic recruitment.

Hematimetric indices, specifically mean corpuscular volume (MCV) and mean corpuscular hemoglobin concentration (MCHC), which evaluate erythrocyte morphological characteristics and mean hemoglobin content, did not exhibit significant variations during either phase of the study. This observation suggests that stimbiotic inclusion, under the conditions examined, did not disrupt erythropoiesis or impair oxygen transport, thereby preserving physiological levels of red blood cell mass.

Total platelet counts, an essential parameter related to hemostatic function, also remained stable with the inclusion of additives during both the initial and growth phases. This finding implies that stimbiotic inclusion did not interfere with primary coagulation mechanisms or physiological platelet activation in European quail (*Coturnix coturnix coturnix*).

In Table 3, concerning the initial phase (days 1 to 14), a significant variation in platelet counts was observed in relation to dietary fiber content, with higher fiber levels correlating with lower platelet counts. Platelets play a critical role in maintaining blood hemostasis through the release of thromboplastin and other factors [34]. The association between a higher-fiber diet and reduced platelet counts may be attributable to enhanced intestinal health, which promotes nutrient absorption in an intestinal environment enriched with beneficial bacteria stimulated by the presence of fiber. Paul et al. [35] indicated that platelets constitutively express transcripts for both pro- and anti-inflammatory cytokines, suggesting that elevated platelet levels in low-fiber diets may be linked to an inflammatory response elicited by dietary composition.

Regarding leukocyte counts, variations were observed in both experimental phases, with a consistent pattern of increased indices in the groups that were not supplemented with the stimbiotic. This response may be associated with heightened exposure to physiological and environmental stress, as changes in total leukocyte numbers in avian species are commonly linked to factors such as management practices, transportation, subclinical infections, and adverse environmental conditions [32]. Thus, the data suggest that animals supplemented with the stimbiotic experienced reduced metabolic stress, potentially due to enhanced intestinal integrity and improved nutrient absorption.

Lymphocyte counts exhibited significant variation across both experimental phases, with a higher proportion of these cells detected in groups that did not receive a diet supplemented with the stimbiotic. Lymphocyte populations in birds can be influenced by a multitude of factors, encompassing physiological, pathological, nutritional, and environmental considerations, as these cells represent the predominant leukocyte population in the peripheral blood of avian species [36] and play a critical role in the adaptive immune response. A comparative analysis of the results from the two experimental phases revealed that lymphocyte counts were lower in animals receiving the stimbiotic. This reduction may indicate an improved immunological status and a more effective response to pathogenic challenges, thereby underscoring the potential modulatory effect of the stimbiotic on the immune system.

Heterophile counts in birds are subject to variation due to numerous physiological, pathological, and environmental factors and are regarded as significant indicators of the immunological and health status of avian species. Notable changes were observed, with animals receiving the stimbiotic during the initial phase displaying lower heterophile values than those that did not receive the supplement. As noted by Markowiak and Śliżewska [37], the gastrointestinal tract serves a critical immunological function and constitutes a primary barrier that protects the host from toxins, pathogens, and their inflammatory effects. The presence of a beneficial microbiota, promoted by the stimbiotic, may mitigate pathogen colonization, reduce the necessity for exaggerated inflammatory responses, and ultimately stabilize or lower heterophile counts under healthy conditions.

Regarding leukocytes, the mean values of eosinophils, basophils, and monocytes did not exhibit significant differences between the experimental groups during the two analyzed phases. The heterophil-to-lymphocyte (H/L) ratio, commonly utilized as a biomarker for stress in avian species [38], also revealed no significant variation between treatments. Given that elevated values of this ratio indicate a heightened level of stress in birds, these findings imply that the stimbiotic, at the dosages and duration administered, did not produce a substantial modulatory effect on the stress response, at least not to a degree sufficient to alter this ratio between treatments.

In the assessment of biochemical parameters, it is essential to acknowledge that these indicators serve as critical reflections of animal physiology, offering insights into organ function, adaptability to nutritional, physiological, and environmental challenges, and aiding in the identification of metabolic imbalances and pathologies [20]. According to [32], investigations focusing on the hepatic system in avian species remain relatively limited, with a notable scarcity of data concerning reference values for these parameters as well as the sensitivity and specificity of the associated enzymes.

An analysis of Table 5 regarding the initial phase indicates that the sole variable exhibiting a significant change was triglycerides. Their concentrations can fluctuate based on sex, diet, and hormonal factors [20], particularly considering the susceptibility of birds to elevated cortisol levels, especially when subjected to stress from captive rearing systems and social grouping. Rezende et al. [39] reported triglyceride levels in broiler chickens ranging from 128.9 to 140.2 mg/dL, while Evans et al. [40] and Silva et al. [41] documented values between 136 and 166 mg/dL. The values obtained in this study were lower than these referenced parameters, potentially attributable to species and age differences.

In the initial phase (1–14 days), the incorporation of various fiber profiles into the diet significantly affected serum triglyceride concentrations, with the stimbiotic demonstrating a notable impact solely on this variable. High-fiber diets, particularly those derived from whole wheat (100H) or predominantly wheat (75H:25L, 50H:50L), resulted in lower triglyceride levels compared to the control diet and the 100% corn formulation (100L). This reduction can be attributed to the higher proportion of soluble fiber found in wheat, which enhances chyme viscosity, prolongs gastric emptying, and diminishes lipid absorption, as well as potentially interfering with micellization and fatty acid absorption in the small intestine [42,43].

The hypolipidemic effect of soluble fiber may also be linked to increased sequestration and excretion of bile acids, compelling the liver to utilize cholesterol for the synthesis of new bile salts, thereby indirectly influencing plasma lipid metabolism [42].

The administration of the stimbiotic resulted in elevated mean serum triglyceride levels compared to diets devoid of the additive, irrespective of the fiber profile. This increase may be associated with the modulatory effects of the stimbiotic on intestinal microbiota, which enhances the fermentation of undigested carbohydrates and elevates the production of short-chain fatty acids, particularly acetate, which serves as a precursor for hepatic lipogenesis in avian species [44].

Although no significant interaction was noted for triglycerides, the primary effects suggest that both fiber composition and the presence of the stimbiotic independently influenced the modulation of this variable [45]. The other biochemical parameters (albumin, AST, GGT, total protein, and cholesterol) did not exhibit statistically significant differences, with the exception of ALT, which displayed a significant interaction, indicating the sensitivity of this enzyme to the specific combination of fiber profile and additive.

These findings reinforce the modulatory role of dietary fiber—particularly wheat—on lipid metabolism in juvenile quails, likely associated with the immaturity of the gastrointestinal tract at this developmental stage, rendering the animals more responsive to alterations in dietary composition [43].

A direct relationship was also observed between a high-fiber diet and increased eosinophil counts in birds during the initial phase. This observation contrasts with low-fiber diets, which exhibited lower eosinophil counts. Research conducted by Cīrule et al. [46] suggests that this variation may be linked to stress, as eosinophil numbers tend to decline under stressful conditions. The underlying mechanism posits that fibers, when hydrolyzed into xylo-oligosaccharides, encourage microbial fermentation and the production of volatile fatty acids. As detailed by Parra et al. [16], these acids enhance digestion and nutrient absorption. This improvement in the digestive process may mitigate stress in the animals, thereby elucidating the higher eosinophil levels observed in high-fiber diets.

When assessing the interaction with the ALT variable, it is crucial to recognize that dietary fiber plays a significant role in the metabolic and digestive health of avian species. In the case of the European quails analyzed, fiber sources such as wheat and corn possess attributes that directly influence ALT levels. Interpreting the increase in serum ALT concentration presents challenges, as it may stem from tissue damage [47] or from physiological variations, such as aging, which can elevate ALT values [32].

Wheat fiber, primarily composed of soluble and insoluble fractions, exhibits a higher fermentation capacity in the intestine, potentially resulting in the production of volatile fatty acids that are advantageous to the intestinal mucosa and overall metabolism. According to Faria et al. [20], avian species with significant liver damage may present normal or even reduced ALT values, signifying that the enzymatic activity of ALT in the hepatic tissue of certain species may be diminished. Most avian species demonstrate serum ALT values ranging from 19 to 50 IU/L [48,49].

**Figure 4:**
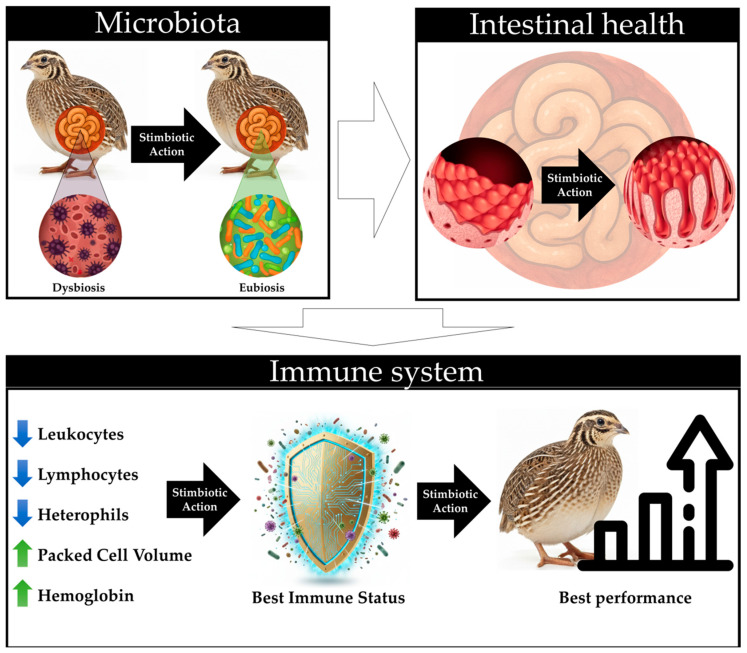
Schemes of stimbiotic effects in quails, showing positive impacts on microbiota, gut health, immunity, and performance.

Previous studies have indicated that inclusion with stimbiotics enhances productive performance, feed efficiency, intestinal microbiota composition, and gut health in broiler chickens [9,14,15,21,22]. Although the present study did not directly assess these parameters, the hematological and biochemical findings suggest an enhancement in the immunological status of quails, as evidenced by reductions in leukocytes, lymphocytes, and heterophils, alongside increases in packed cell volume and hemoglobin. These results imply that the stimbiotic likely induced effects similar to those reported in prior studies, fostering a healthier intestinal environment, optimizing nutrient absorption, and enhancing the physiological and immunological balance in the birds (Figure 4).

The findings of the present study indicate that inclusion with a stimbiotic and variations in fiber profiles independently modulated hematological and biochemical parameters in broiler quails. The inclusion of the stimbiotic resulted in increases in packed cell volume and hemoglobin levels, alongside reductions in leukocytes, lymphocytes, and heterophils, which suggest a decrease in physiological stress and an improvement in immunological balance. Conversely, the introduction of different fiber profiles independently affected specific variables, such as platelet counts and triglyceride levels, thereby underscoring their significance in intestinal and lipid metabolism. Although productive performance and gut health were not explicitly assessed, the results imply that the stimbiotic may have elicited effects analogous to those previously documented in broiler chickens, enhancing nutrient absorption, physiological equilibrium, and the maintenance of a healthy immunological status in the subjects.

## 5. Conclusions

The application of the stimbiotic is recommended during both the initial and growth phases, as it shows a beneficial impact on the avian immune system. Conversely, high-fiber diets provide enhanced advantages primarily during the growth phase.

## Figures and Tables

**Table 1:** Composition and nutritional values of diets with different fiber profiles, with or without stimbiotic, for European quails in the starter phase (1–14 days).

Ingredients, %	With Stimbiotic						Without Stimbiotic					
	Control	100H	75H25L	50H50L	25H75L	100L	Control	100H	75H25L	50H50L	25H75L	100L
Corn	49.885	0.000	11.723	23.446	35.169	46.892	49.885	0.000	11.723	23.446	35.169	46.892
Soybean meal	45.331	39.758	40.913	42.068	43.223	44.378	45.331	39.758	40.913	42.068	43.223	44.378
Whole wheat	0.000	16.799	12.599	8.399	4.200	0.000	0.000	16.799	12.599	8.399	4.200	0.000
Wheat bran	0.000	30.337	22.753	15.169	7.584	0.000	0.000	30.337	22.753	15.169	7.584	0.000
Corn gluten	0.000	0.000	0.804	1.607	2.411	3.215	0.000	0.000	0.804	1.607	2.411	3.215
Soybean oil	1.544	10.100	8.145	6.190	4.235	2.280	1.544	10.100	8.145	6.190	4.235	2.280
Dicalcium phosphate	1.053	0.384	0.544	0.704	0.864	1.023	1.053	0.384	0.544	0.704	0.864	1.023
Limestone	1.110	1.484	1.395	1.306	1.218	1.129	1.110	1.484	1.395	1.306	1.218	1.129
Salt	0.393	0.389	0.386	0.384	0.382	0.379	0.393	0.389	0.386	0.384	0.382	0.379
L-Lysine	0.098	0.153	0.143	0.134	0.125	0.116	0.098	0.153	0.143	0.134	0.125	0.116
DL-Methionine	0.373	0.385	0.382	0.379	0.376	0.373	0.373	0.385	0.382	0.379	0.376	0.373
L-Threonine	0.000	0.000	0.000	0.000	0.000	0.000	0.000	0.000	0.000	0.000	0.000	0.000
Mineral premix ^1^	0.150	0.150	0.150	0.150	0.150	0.150	0.150	0.150	0.150	0.150	0.150	0.150
Vitamin premix ^2^	0.050	0.050	0.050	0.050	0.050	0.050	0.050	0.050	0.050	0.050	0.050	0.050
Quantum Blue ^3^	0.003	0.003	0.003	0.003	0.003	0.003	0.003	0.003	0.003	0.003	0.003	0.003
Signis ^4^	0.010	0.010	0.010	0.010	0.010	0.010	0.000	0.000	0.000	0.000	0.000	0.000
Inert	0.000	0.000	0.000	0.000	0.000	0.000	0.010	0.010	0.010	0.010	0.010	0.010
Total	100.000	100.000	100.000	100.000	100.000	100.000	100.000	100.000	100.000	100.000	100.000	100.000
Calculated nutrients												
CP, %	25	25	25	25	25	25	25	25	25	25	25	25
ME, kcal/kg	2900	2900	2900	2900	2900	2900	2900	2900	2900	2900	2900	2900
CF, %	2.808	4.855	4.386	3.916	3.446	2.977	2.808	4.855	4.386	3.916	3.446	2.977
NDF, %	13.049	19.383	17.960	16.538	15.115	13.693	13.049	19.383	17.960	16.538	15.115	13.693
MM, %	3.333	4.052	3.895	3.738	3.582	3.425	3.333	4.052	3.895	3.738	3.582	3.425
Starch, %	34.519	21.041	24.075	27.110	30.144	33.178	34.519	21.041	24.075	27.110	30.144	33.178
Total NSPs, %	10.269	15.100	14.031	12.961	11.892	10.822	10.269	15.100	14.031	12.961	11.892	10.822
Insoluble NSPs, %	8.365	12.181	11.354	10.527	9.699	8.872	8.365	12.181	11.354	10.527	9.699	8.872
Soluble NSPs, %	1.904	2.919	2.677	2.434	2.192	1.950	1.904	2.919	2.677	2.434	2.192	1.950
Total AX, %	3.405	6.749	5.993	5.238	4.483	3.727	3.405	6.749	5.993	5.238	4.483	3.727
Soluble AX, %	0.327	0.900	0.762	0.625	0.487	0.350	0.327	0.900	0.762	0.625	0.487	0.350
Insoluble AX, %	3.078	5.847	5.230	4.612	3.995	3.377	3.078	5.847	5.230	4.612	3.995	3.377
EE, %	4.731	12.495	10.726	8.957	7.189	5.420	4.731	12.495	10.726	8.957	7.189	5.420
Calcium, %	0.850	0.850	0.850	0.850	0.850	0.850	0.850	0.850	0.850	0.850	0.850	0.850
Available phosphorus, %	0.320	0.320	0.320	0.320	0.320	0.320	0.320	0.320	0.320	0.320	0.320	0.320
Sodium, %	0.170	0.170	0.170	0.170	0.170	0.170	0.170	0.170	0.170	0.170	0.170	0.170
Chlorine, %	0.302	0.286	0.289	0.292	0.295	0.297	0.302	0.286	0.289	0.292	0.295	0.297
Potassium, %	0.989	1.127	1.094	1.060	1.027	0.994	0.989	1.127	1.094	1.060	1.027	0.994
Digestible amino acids												
Lysine, %	1.370	1.370	1.370	1.370	1.370	1.370	1.370	1.370	1.370	1.370	1.370	1.370
Methionine, %	0.703	0.692	0.695	0.697	0.699	0.702	0.703	0.692	0.695	0.697	0.699	0.702
Methionine + Cysteine, %	1.040	1.040	1.040	1.040	1.040	1.040	1.040	1.040	1.040	1.040	1.040	1.040
Threonine, %	0.856	0.787	0.803	0.819	0.835	0.852	0.856	0.787	0.803	0.819	0.835	0.852
Tryptophan, %	0.297	0.306	0.303	0.300	0.296	0.293	0.297	0.306	0.303	0.300	0.296	0.293
Valine, %	1.057	1.020	1.029	1.038	1.048	1.057	1.057	1.020	1.029	1.038	1.048	1.057

^1^ Copper 10,000 mg; Iodine 160 mg; Manganese 14,000 mg; Selenium 108 mg and Zinc 14,000 mg; ^2^ Mineral and vitamin premix: Levels per kg of product: Vit. at 2,090,000 IU; Vit. E 7600 mg; Vit D3 332,500 IU; Vit k3 950 mg; Nicotinic Acid 8500 mg; Vit B1 475 mg; Vit B12 3800 mg; Vit. B2 1900 mg; Folic Acid 237.5 mg; Biotin 38 mb; Choline 72,000 mg; Pantothenic Acid 3800 mg; ^3^ Phytase equivalent to the supplemental dose of 500 FTU/kg of feed; ^4^ Stimbiotic: β-1,4-endo-xylanase and xylo-oligosaccharides, AB Vista, Marlborough, UK, providing 16,000 BXU/kg; L100 = 100% high fiber—whole wheat and wheat bran; L75H25 = 75% high fiber + 25% low fiber; L50H50 = 50% high fiber + 50% low fiber; L25H75 = 25% high fiber + 75% low fiber; H100 = 100% low fiber—corn germ; CP = Crude Protein; ME = Metabolizable Energy; CF = Crude Fiber; NDF = Neutral Detergent Fiber; MM = Mineral Matter; NSPs = Non-Starch Polysaccharides; AX = Arabinoxylans; EE = Ether Extract.

**Table 2:** Composition and nutritional values of diets with different fiber profiles, with or without stimbiotic, for European quails in the growth phase (15–35 days).

Ingredients, %	With Stimbiotic						Without Stimbiotic					
	Control	100H	75H25L	50H50L	25H75L	100L	Control	100H	75H25L	50H50L	25H75L	100L
Corn	60.491	0.000	14.245	28.490	42.735	56.980	60.491	0.000	14.245	28.490	42.735	56.980
Soybean meal	34.809	28.153	29.773	31.393	33.012	34.632	34.809	28.153	29.773	31.393	33.012	34.632
Whole wheat	0.000	36.122	27.091	18.061	9.030	0.000	0.000	36.122	27.091	18.061	9.030	0.000
Wheat bran	0.000	3.761	2.821	1.880	0.940	0.000	0.000	3.761	2.821	1.880	0.940	0.000
Corn gluten	0.000	19.834	15.613	11.392	7.171	2.950	0.000	19.834	15.613	11.392	7.171	2.950
Soybean oil	2.055	9.628	7.929	6.231	4.532	2.833	2.055	9.628	7.929	6.231	4.532	2.833
Dicalcium phosphate	0.868	0.534	0.609	0.685	0.760	0.835	0.868	0.534	0.609	0.685	0.760	0.835
Limestone	0.937	1.114	1.074	1.033	0.993	0.953	0.937	1.114	1.074	1.033	0.993	0.953
Salt	0.345	0.263	0.280	0.298	0.315	0.333	0.345	0.263	0.280	0.298	0.315	0.333
L-Lysine	0.000	0.092	0.069	0.046	0.023	0.000	0.000	0.092	0.069	0.046	0.023	0.000
DL-Methionine	0.223	0.187	0.194	0.202	0.210	0.217	0.223	0.187	0.194	0.202	0.210	0.217
L-Threonine	0.059	0.100	0.088	0.077	0.065	0.054	0.059	0.100	0.088	0.077	0.065	0.054
Mineral premix ^1^	0.150	0.150	0.150	0.150	0.150	0.150	0.150	0.150	0.150	0.150	0.150	0.150
Vitamin premix ^2^	0.050	0.050	0.050	0.050	0.050	0.050	0.050	0.050	0.050	0.050	0.050	0.050
Quantum Blue ^3^	0.003	0.003	0.003	0.003	0.003	0.003	0.003	0.003	0.003	0.003	0.003	0.003
Signis ^4^	0.010	0.010	0.010	0.010	0.010	0.010	0.000	0.000	0.000	0.000	0.000	0.000
Inert	0.000	0.000	0.000	0.000	0.000	0.000	0.010	0.010	0.010	0.010	0.010	0.010
Total	100.000	100.000	100.000	100.000	100.000	100.000	100.000	100.000	100.000	100.000	100.000	100.000
Calculated nutrients												
CP, %	22	22	22	22	22	22	22	22	22	22	22	22
ME, kcal/kg	3050	3050	3050	3050	3050	3050	3050	3050	3050	3050	3050	3050
CF, %	2.540	4.020	3.693	3.366	3.039	2.712	2.540	4.020	3.693	3.366	3.039	2.712
NDF, %	13.084	16.736	15.968	15.200	14.432	13.664	13.084	16.736	15.968	15.200	14.432	13.664
MM, %	2.805	3.611	3.439	3.268	3.096	2.925	2.805	3.611	3.439	3.268	3.096	2.925
Starch, %	40.580	27.205	30.131	33.056	35.982	38.908	40.580	27.205	30.131	33.056	35.982	38.908
Total NSPs, %	9.017	14.000	12.901	11.803	10.704	9.605	9.017	14.000	12.901	11.803	10.704	9.605
Insoluble NSPs, %	7.555	11.641	10.748	9.856	8.964	8.071	7.555	11.641	10.748	9.856	8.964	8.071
Soluble NSPs, %	1.462	2.359	2.153	1.946	1.740	1.534	1.462	2.359	2.153	1.946	1.740	1.534
Total AX, %	3.322	6.380	5.691	5.001	4.312	3.622	3.322	6.380	5.691	5.001	4.312	3.622
Soluble AX, %	0.275	0.800	0.675	0.550	0.425	0.300	0.275	0.800	0.675	0.550	0.425	0.300
Insoluble AX, %	3.047	5.577	5.013	4.450	3.886	3.322	3.047	5.577	5.013	4.450	3.886	3.322
EE, %	5.344	11.705	10.296	8.887	7.478	6.070	5.344	11.705	10.296	8.887	7.478	6.070
Calcium, %	0.700	0.700	0.700	0.700	0.700	0.700	0.700	0.700	0.700	0.700	0.700	0.700
Available phosphorus, %	0.270	0.270	0.270	0.270	0.270	0.270	0.270	0.270	0.270	0.270	0.270	0.270
Sodium, %	0.150	0.150	0.150	0.150	0.150	0.150	0.150	0.150	0.150	0.150	0.150	0.150
Chlorine, %	0.278	0.244	0.251	0.258	0.266	0.273	0.278	0.244	0.251	0.258	0.266	0.273
Potassium, %	0.831	0.892	0.880	0.868	0.857	0.845	0.831	0.892	0.880	0.868	0.857	0.845
Digestible amino acids												
Lysine, %	1.020	1.020	1.020	1.020	1.020	1.020	1.020	1.020	1.020	1.020	1.020	1.020
Methionine, %	0.510	0.471	0.479	0.488	0.497	0.506	0.510	0.471	0.479	0.488	0.497	0.506
Methionine + Cysteine, %	0.800	0.800	0.800	0.800	0.800	0.800	0.800	0.800	0.800	0.800	0.800	0.800
Threonine, %	0.780	0.780	0.780	0.780	0.780	0.780	0.780	0.780	0.780	0.780	0.780	0.780
Tryptophan, %	0.242	0.236	0.237	0.239	0.240	0.241	0.242	0.236	0.237	0.239	0.240	0.241
Valine, %	0.880	0.920	0.913	0.906	0.899	0.892	0.880	0.920	0.913	0.906	0.899	0.892

^1^ Copper 10,000 mg; Iodine 160 mg; Manganese 14,000 mg; Selenium 108 mg and Zinc 14,000 mg; ^2^ Mineral and vitamin premix: Levels per kg of product: Vit. at 2,090,000 IU; Vit. E 7600 mg; Vit D3 332,500 IU; Vit k3 950 mg; Nicotinic Acid 8500 mg; Vit B1 475 mg; Vit B12 3800 mg; Vit. B2 1900 mg; Folic Acid 237.5 mg; Biotin 38 mb; Choline 72,000 mg; Pantothenic Acid 3800 mg; ^3^ Phytase equivalent to the supplemental dose of 500 FTU/kg of feed; ^4^ Stimbiotic: β-1,4-endo-xylanase and xylo-oligosaccharides, AB Vista, Marlborough, UK, providing 16,000 BXU/kg; L100 = 100% high fiber—whole wheat and wheat bran; L75H25 = 75% high fiber + 25% low fiber; L50H50 = 50% high fiber + 50% low fiber; L25H75 = 25% high fiber + 75% low fiber; H100 = 100% low fiber—corn germ; CP = Crude Protein; ME = Metabolizable Energy; CF = Crude Fiber; NDF = Neutral Detergent Fiber; MM = Mineral Matter; NSPs = Non-Starch Polysaccharides; AX = Arabinoxylans; EE = Ether Extract.

**Table 3:** Hemogram of European quails (*Coturnix coturnix coturnix*) at 14 days of age, fed diets with different fiber profiles, with or without stimbiotic inclusion during the period from 1 to 14 days.

Variables	Stimbiotic		Fiber						SEM	*p*-Value		
	−	+	Control	100H	75H25L	50H50L	25H75L	100L		S	F	S × F
Packed cell volume, L/L	0.341	0.343	0.351	0.344	0.334	0.337	0.342	0.343	2.76	0.6586	0.6129	0.8699
Erythrocyte count, ×10^12^/L	2.49	2.39	2.45	2.41	2.29	2.44	2.46	2.60	0.39	0.2353	0.3883	0.7497
Hemoglobin, g/dL	6.61 b	7.35 a	7.16	7.02	6.86	6.87	6.91	7.08	0.75	<0.001	0.8249	0.2448
MCV, fL	139	145.95	145.37	145.71	151.14	140.45	142.01	132.11	20.30	0.1555	0.2193	0.6958
MCHC, g/dL	19.41	21.04	20.44	20.34	20.52	20.24	20.22	20.63	1.37	0.0564	0.9600	0.0560
Total platelets, ×10^9^/L	39.26	35.96	45.87 a	31.69 b	34.22 b	35.07 b	40.37 ab	36.25 b	10.78	0.0848	0.0062	0.3595
Total leukocyte count, ×10^9^/L	8.81 a	6.70 b	8.07	7.49	6.12	7.42	8.91	8.44	3.69	0.0065	0.3626	0.1383
Monocyte, ×10^9^/L	0.02	0.02	0.04	0.01	0.01	0.01	0.02	0.02	0.05	0.3533	0.4431	0.4219
Lymphocyte, ×10^9^/L	5.85 a	4.79 b	5.25	5.07	4.68	5.11	6.45	5.31	2.48	0.0392	0.4652	0.0797
Eosinophil, ×10^9^/L	0.28	0.28	0.38	0.17	0.15	0.21	0.21	0.31	0.26	0.1043	0.1500	0.4244
Heterophile, ×10^9^/L	2.49 a	1.58 b	2.22	2.04	1.13	1.93	2.11	2.71	1.80	0.0162	0.2735	0.5212
Basophil, ×10^9^/L	0.19	0.17	0.18	0.19	0.15	0.14	0.12	0.08	0.13	0.0509	0.1763	0.3053
H/L	0.43	0.33	0.42	0.40	0.24	0.38	0.33	0.51	0.73	0.5346	0.3642	0.7329

MCV = mean corpuscular volume; MCHC = mean corpuscular hemoglobin concentration; H/L = heterophile/lymphocyte ratio. The symbol (+) indicates inclusion with the stimbiotic and (–) indicates no inclusion. Fiber profiles: Control (corn- and soybean meal-based diet); L100 (100% high fiber—whole wheat and wheat bran); L75H25 (75% high fiber + 25% low fiber); L50H50 (50% high fiber + 50% low fiber); L25H75 (25% high fiber + 75% low fiber); H100 (100% low fiber—corn germ). Different letters in the same row indicate significant differences according to Tukey’s test (*p* < 0.05). SEM = standard error of the mean. S = stimbiotic; F = fiber; S × F = stimbiotic × fiber interaction.

**Table 4:** Hemogram of European quails (*Coturnix coturnix coturnix*) at 35 days of age, fed diets with different fiber profiles, with or without stimbiotic inclusion during the period from 1 to 35 days.

Variables	Stimbiotic		Fiber						SEM	*p*-Value		
	−	+	Control	100H	75H25L	50H50L	25H75L	100L		S	F	S × F
Packed cell volume, L/L	0.403 b	0.428 a	0.423	0.427	0.404	0.401	0.415	0.427	5.15	0.0205	0.6528	0.7959
Erythrocyte count, ×10^12^/L	3.54	3.76	4.41	3.20	3.23	3.13	4.56	3.43	2.94	0.7117	0.5789	0.2184
Hemoglobin, g/dL	9.18 b	9.89 a	9.88	9.69	8.99	9.64	9.60	9.45	1.33	0.0117	0.5370	0.1136
MCV, fL	128.11	127.78	127.42	132.79	127.41	129.64	122.61	127.71	24.82	0.9482	0.9270	0.2774
MCHC, g/dL	22.33	22.66	21.89	23.04	22.31	24.07	21.67	21.97	3.82	0.6817	0.4941	0.1258
Total platelets, ×10^9^/L	35.98	32.79	37.69	38.50	33.66	27.70	33.57	35.16	12.37	0.2022	0.1832	0.8435
Total leukocyte count, ×10^9^/L	9.09 a	7.71 b	7.03	8.90	8.25	9.72	9.13	7.38	3.11	0.0386	0.1256	0.0803
Monocyte, ×10^9^/L	0.16	0.14	0.35	0.03	0.02	0.06	0.41	0.03	0.80	0.9282	0.5604	0.2698
Lymphocyte, ×10^9^/L	6.66 a	5.77 b	5.56	6.02	6.58	7.17	6.11	5.82	2.10	0.0437	0.3156	0.1298
Eosinophil, ×10^9^/L	0.48	0.46	0.30 b	0.78 a	0.34 b	0.57 ab	0.48 ab	0.37 b	0.38	0.8338	0.0055	0.1298
Heterophile, ×10^9^/L	1.74	1.36	1.04	1.76	1.27	1.82	2.43	1.05	1.76	0.2950	0.2155	0.2246
Basophil, ×10^9^/L	0.11	0.11	0.13	0.11	0.04	0.09	0.18	0.11	0.20	0.9000	0.5426	0.5995
H/L	0.26	0.24	0.19	0.29	0.19	0.25	0.40	0.18	0.84	0.6100	0.5350	0.9460

MCV = mean corpuscular volume; MCHC = mean corpuscular hemoglobin concentration; H/L = heterophile/lymphocyte ratio. The symbol (+) indicates inclusion with the stimbiotic and (–) indicates no inclusion. Fiber profiles: Control (corn- and soybean meal-based diet); L100 (100% high fiber—whole wheat and wheat bran); L75H25 (75% high fiber + 25% low fiber); L50H50 (50% high fiber + 50% low fiber); L25H75 (25% high fiber + 75% low fiber); H100 (100% low fiber—corn germ). Different letters in the same row indicate significant differences according to Tukey’s test (*p* < 0.05). SEM = standard error of the mean. S = stimbiotic; F = fiber; S × F = stimbiotic × fiber interaction.

**Table 5:** Biochemical parameters of European quails (*Coturnix coturnix coturnix*) at 14 days of age, fed diets with different fiber profiles, with or without stimbiotic inclusion during the period from 1 to 14 days.

Variables	Stimbiotic		Fiber						SEM	*p*-Value		
	−	+	Control	100H	75H25L	50H50L	25H75L	100L		S	F	S × F
Albumin, g/dL	1.14	1.1	1.17	1.01	1.16	1.13	1.14	1.13	0.15	0.2573	0.0652	0.636
ALT, UI/L	10.58	14.38	14.6	13.19	13.19	11.81	11.13	10.94	4.52	0.5768	0.1662	0.0083
AST, UI/L	218.71	214.42	230.08	224.25	218.81	215.88	215.69	194.5	58.63	0.7208	0.6337	0.4337
GGT, UI/L	1.29	1.27	1.25	1.29	1.71	1.46	1.15	0.86	1.19	0.9018	0.4749	0.3318
Total protein, g/dL	2.94	2.88	2.98	2.76	2.96	2.95	2.91	2.94	0.34	0.3248	0.5286	0.2842
Cholesterol, mg/dL	149.45	140.68	136.08	142.74	157.26	152.46	146.29	138.56	29.22	0.105	0.2962	0.6456
Triglycerides, mg/dL	77.51 b	115.58 a	144.86 a	73.36 b	72.82 b	93.84 b	104.84 ab	90.07 b	46.91	0.0001	0.0003	0.3197

ALT = alanine aminotransferase; AST = aspartate aminotransferase; GGT = gamma-glutamyl transferase. The symbol (+) indicates inclusion with the stimbiotic and (–) indicates no inclusion. Fiber profiles: Control (corn- and soybean meal-based diet); L100 (100% high fiber—whole wheat and wheat bran); L75H25 (75% high fiber + 25% low fiber); L50H50 (50% high fiber + 50% low fiber); L25H75 (25% high fiber + 75% low fiber); H100 (100% low fiber—corn germ). Different letters in the same row indicate significant differences according to Tukey’s test (*p* < 0.05). SEM = standard error of the mean. S = stimbiotic; F = fiber; S × F = stimbiotic × fiber interaction.

**Table 6:** Biochemical parameters of European quails (*Coturnix coturnix coturnix*) at 35 days of age, fed diets with different fiber profiles, with or without stimbiotic inclusion during the period from 1 to 35 days.

Variables	Stimbiotic		Fiber						SEM	*p*-Value		
	−	+	Control	100H	75H25L	50H50L	25H75L	100L		S	F	S × F
**Albumin, g/dL**	5.61	4.94	13.89	1.24	1.36	1.40	12.65	1.31	26.91	0.9048	0.5368	0.2791
ALT, UI/L	12.72	12.625	11.74	9.84	13.75	16.50	12.41	11.88	12.99	0.9393	0.7968	0.4774
AST, UI/L	198.69	212.96	206.18	204.79	201.88	199.44	199.86	222.13	52.29	0.1868	0.3386	0.0774
GGT, UI/L	37.72	89.94	15.10	36.53	45.07	137.76	94.89	53.89	139.21	0.0726	0.1520	0.9620
Total protein, g/dL	3.35	3.63	3.35	3.20	3.50	3.70	3.88	3.30	0.84	0.1013	0.1995	0.3563
Cholesterol, mg/dL	186.54	195.79	199.75	169.79	182.72	208.11	188.26	196.86	63.10	0.4795	0.6011	0.4710
Triglycerides, mg/dL	285.94	216.99	190.46	244.43	294.29	158.16	324.71	300.87	244.26	0.1749	0.3409	0.2480

ALT = alanine aminotransferase; AST = aspartate aminotransferase; GGT = gamma-glutamyl transferase. The symbol (+) indicates inclusion with the stimbiotic and (–) indicates no inclusion. Fiber profiles: Control (corn- and soybean meal-based diet); L100 (100% high fiber—whole wheat and wheat bran); L75H25 (75% high fiber + 25% low fiber); L50H50 (50% high fiber + 50% low fiber); L25H75 (25% high fiber + 75% low fiber); H100 (100% low fiber—corn germ). SEM = standard error of the mean. S = stimbiotic; F = fiber; S × F = stimbiotic × fiber interaction.

**Table 7:** Performance of European quails (*Coturnix coturnix coturnix*) fed diets with different fiber profiles, with or without stimbiotic inclusion during the period from 1 to 35 days of age.

Variables	Stimbiotic		Fiber						SEM	*p*-Value		
	−	+	Control	100H	75H25L	50H50L	25H75L	100L		S	F	S × F
Initial Body Weight, g	10.17	10.18	10.17	10.17	10.18	10.17	10.18	10.18	0.006	0.7343	0.9889	0.9403
Body Weight, g	331.64	347.48	343.93	307.24	331.21	316.53	377.18	358.86	8.664	0.3629	0.1902	0.7157
Daily Feed Intake, g/bird	18.67 a	17.85 b	18.21 ab	18.01 ab	17.17 b	17.85 ab	19.25 a	18.87 ab	0.204	0.0303	0.0423	0.1784
Total Feed Intake, g/bird	653.40 a	624.90 b	637.30 ab	630.30 ab	601.06 b	624.74 ab	673.90 a	660.50 ab	7.136	0.0303	0.0423	0.1784
Daily Weight Gain, g/bird	9.18	9.64	9.54	8.49	9.17	8.75	10.49	9.96	0.248	0.3630	0.1903	0.7157
Total Weight Gain, g/bird	321.46	337.30	333.76	297.07	321.03	306.36	367.00	348.68	8.664	0.3630	0.1903	0.7157
Feed Conversion Ratio, g/g	2.10 b	1.91 a	1.97	2.17	1.93	2.07	1.95	1.91	0.047	0.0491	0.5552	0.4168
Carcass Yield, %	79.48	79.74	76.41	80.42	81.04	78.83	80.32	80.11	0.622	0.839	0.377	0.616

The symbol (+) indicates inclusion with the stimbiotic and (–) indicates no inclusion. Fiber profiles: Control (corn- and soybean meal-based diet); L100 (100% high fiber—whole wheat and wheat bran); L75H25 (75% high fiber + 25% low fiber); L50H50 (50% high fiber + 50% low fiber); L25H75 (25% high fiber + 75% low fiber); H100 (100% low fiber—corn germ). Different letters in the same row indicate significant differences according to Tukey’s test (*p* < 0.05). SEM = standard error of the mean. S = stimbiotic; F = fiber; S × F = stimbiotic × fiber interaction.

## Data Availability

Dataset available on request from the authors.
